# Dual Function of CCAT2 in Regulating Luminal Subtype of Breast Cancer Depending on the Subcellular Distribution

**DOI:** 10.3390/cancers15020538

**Published:** 2023-01-16

**Authors:** Heying Xie, Yuefan Guo, Zhen Xu, Qiong Wang, Tao Wang, Yi Gu, Danni Li, Yu Liu, Wenjing Ma, Pengfei Liu, Qian Zhao, Jinhui Lü, Junjun Liu, Zuoren Yu

**Affiliations:** 1 Shanghai East Hospital, Jinzhou Medical University, Shanghai 200120, China; 2 Research Center for Translational Medicine, Shanghai East Hospital, Tongji University School of Medicine, Shanghai 200120, China; 3 Affiliated Hospital of Nantong University, Nantong 226001, China

**Keywords:** CCAT2, luminal breast cancer, cancer stem cell, cell proliferation

## Abstract

**Simple Summary:**

Long non-coding RNAs have been demonstrated to play important roles in regulating tumor development and progression in breast cancer, which is tumor type dependent and cellular localization dependent. However, the regulatory mechanisms remain unclear. Herein, we found a dual function of long non-coding RNA CCAT2 in the luminal subtype of breast cancer, depending on its subcellular distribution. CCAT2 showed an overall downregulation in the tumor tissues from the luminal breast cancer patients. Cytoplasmic CCAT2 in the luminal subtype of breast cancer cell MCF-7 or T47D significantly suppressed cell proliferation and cancer cell stemness in vitro. It inhibited tumor growth in vivo, which was mediated with miR-221-p27 signaling. In contrast, nuclear overexpression of CCAT2 led to upregulation of OCT4-PG1 and the induction of cancer cell stemness. In summary, for the first time, the current study revealed a dual function of lncRNA CCAT2 as a tumor suppressor or oncogene depending upon its subcellular distribution.

**Abstract:**

Breast cancer is the most common cancer in women around the world. Emerging evidence has indicated the important roles that non-coding RNAs play in regulating tumor development and progression in breast cancer. Herein, we found a dual function of long non-coding RNA (LncRNA) CCAT2 in the luminal subtype of breast cancer, depending on its subcellular distribution. CCAT2 showed an overall downregulation in the tumor tissues from luminal breast cancer patients. Transient overexpression of CCAT2 in the luminal subtype of breast cancer cell MCF-7 or T47D significantly suppressed cell proliferation in vitro and inhibited tumor growth in vivo. Gene expression analysis of cancer stem cell markers including OCT4, NANOG, h-TERT, SOX2 and KLF4; flow cytometry analysis of breast cancer stem cell population, and mammosphere formation assay demonstrated inhibition of cancer cell stemness with transient transfection of CCAT2 in which exogenous CCAT2 mainly distributed in the cytoplasm and regulated miR-221-p27 signaling via RNA sequence interaction. However, overexpression of CCAT2 in MCF-7 cells through pMX retroviral nuclear expression vector accumulated CCAT2 in the nucleus, leading to upregulation of OCT4-PG1, a pseudogene of stem gene OCT4, thereby promoting the cancer cell stemness. In conclusion, the current study, for the first time, revealed a dual function of lncRNA CCAT2 as a tumor suppressor or oncogene depending upon its subcellular distribution. It also demonstrated the regulatory mechanism of cytoplasmic CCAT2 in suppressing tumorigenesis in the luminal subtype of breast cancer.

## 1. Introduction

According to the 2022 Health Report from the American Cancer Society, breast cancer accounts for nearly one-third of new cancer cases and 15% of cancer-related deaths in women [1]. Although the progress in diagnosing and treating breast cancer patients has greatly improved the 10-year survival rate, the incidence rate is still rising, and metastatic breast cancer remains incurable [2].

Clinically, breast cancer is classified into four common subtypes, including luminal A, luminal B, human epidermal growth factor receptor 2 (HER2)-enriched and basal-like breast cancer [3]. About ~70% of breast cancer belongs to the luminal subtype with response to hormone therapy, and ~15% belong to basal-like (mostly triple-negative breast cancer (TNBC)) with frequent metastasis and poor survival [4]. Currently, drug resistance, tumor metastasis and tumor relapse of breast cancer are becoming the biggest challenges to clinical doctors and research scientists. These are all related to a small population of cells within tumors, termed cancer stem cells (CSCs) [5]. CSCs hide in tumor tissues as “seeds” with a strong ability to spring forth to regenerate tumors [6]. The molecular mechanisms regulating CSCs need to be investigated, and novel therapeutic strategies targeting CSCs in the treatment of breast cancer should be developed.

Emerging evidence has indicated the important roles that long non-coding RNAs (lncRNAs) play in regulating tumorigenesis and CSCs in breast cancer [7,8,9,10]. LncRNAs are a class of non-coding RNAs with over 200 nucleotides in length [11,12]. LncRNAs usually act as decoy or competitive endogenous RNA (ceRNA) in gene expression regulation by interacting with transcription factors, RNA-binding proteins, or small non-coding RNAs [13,14,15]. In breast cancer, LINC01615 [8], the long non-coding NKILA [9] and MALINC1 [10] have been reported to regulate breast cancer progression and metastasis. Notably, the function of a lncRNA is closely related to its subcellular localization [16]. Most lncRNAs are located in the nucleus to regulate gene transcription at transcriptional levels [17]. Some lncRNAs are located in the cytoplasm, mediating signal transduction pathways and/or regulating gene transcription at post-transcriptional levels [18]. For example, LINC01133 was found in the cytoplasm of gastric cancer cells, interacting with miR-106a-3p to inactivate Wnt signaling and inhibit gastric cancer metastasis [19]. LncRNA CASC21 induced HGH1 expression by recruiting POU5F1B to the HGH1 promoter in the nucleus and sponging miR-485-5p in the cytoplasm, thereby facilitating colorectal cancer cell proliferation, migration and stemness [20].

CCAT2 was originally discovered by Ling, et al., in 2013 in colon cancer [21] and then in multiple other types of cancers, including gastric cancer [22], cervical cancer [23] and breast cancer [24]. Our previous study found an upregulation of CCAT2 in triple-negative breast cancer [25]. Different from the triple negative subtype, herein, we showed the downregulation of CCAT2 in luminal breast cancer and found a positive correlation between the expression levels of CCAT2 and overall survival rate in the luminal subtype of breast cancer patients. Transient overexpression of CCAT2 in MCF-7 or T47D cells significantly suppressed cell proliferation and cell stemness. In mechanism, we found a greater distribution of CCAT2 in the cytoplasm of luminal breast cancer cells than in TNBC cells. Transient transfection of CCAT2 increased the cytoplasmic localization of CCAT2, which sponged miR-221/222 through sequence complementarity, rescued the expression inhibition of p27 by miR-221/222, and thereby inhibited tumor growth. However, transfection of CCAT2 using pMX retroviral nuclear expression vector only accumulated CCAT2 in the nucleus, leading to upregulation of OCT4-PG1, which positively regulated cancer cell stemness. Our study revealed a dual function of CCAT2 in the luminal subtype of breast cancer depending on its subcellular distribution, which will help improve our understanding of molecular mechanisms for breast cancer regulation by CCAT2.

## 2. Materials and Methods

### 2.1. Tumor Samples

Luminal subtype of tumor samples from breast cancer patients was collected from Tongji University School of Medicine, Shanghai East Hospital. The project was approved by the Institutional Review Board (IRB) of Shanghai East Hospital with the ethical approval number #2020-YanShen-155.

### 2.2. Animals

The 6~8-week-old female BALB/c nude mice were purchased from the Silaike company (Shanghai, China). The animal experiments were performed following all the regulations from the Institutional Animal Care and Use Committee, Tongji University School of Medicine.

### 2.3. Cell Lines and Cell Culture

The breast cancer cell lines MCF-7 and T-47D were originally purchased from ATCC and regularly maintained in our lab. These cells were cultured in Dulbecco’s Modified Eagle Medium (DMEM) containing 100 mg/L penicillin and streptomycin, 10% fetal bovine serum (FBS), and cultured at 37 °C and 5% CO_2_.

### 2.4. Vectors

CCAT2-pMX vector, CCAT2-pcDNA 3.1 vector and controls were presented by Dr. George A. Calin at MD Anderson Cancer Center, University of Texas.

### 2.5. First Strand cDNA Preparation and Real-Time PCR

Trizol reagent (Invitrogen, Carlsbad, CA, USA) was used to extract total RNA. An mRNA reverse transcription was performed using a regular approach and random primer. The M&G miRNA Reverse Transcription kit (miRGenes, Shanghai, China) was used to prepare cDNAs of small RNAs according to the manufacturer’s instructions. Real-time PCR assays were conducted using SYBR Green Master Mix (Applied Biosystem, Thermo Fisher Scientific, Foster, CA, USA) with QuantStudio 6.0 Sequence Detection System (Applied Biosystem, Foster, CA, USA). β-actin or GAPDH was used for mRNA normalization, and a 5S ribosome RNA was used for miRNA normalization.

### 2.6. Cell Proliferation and Colony Formation Assay

Cell Counting Kit-8 (CCK-8) and colony formation assays were applied to determine cell proliferation. Briefly, 1.5 × 10^3^ cells/well were seeded in a 96-well plate for CCK8 analysis. At the indicated time, 10 ul/well of CCK-8 reagent was added to incubate for 3 h, followed by absorbance detection at 450 nm. For the colony formation assay, 1.5 × 10^3^ cells were seeded in a 12-well plate and cultured for 7–10 days to form colonies. After fixing with 4% paraformaldehyde and staining with 0.5% crystal violet, colonies were quantitated under a microscope.

### 2.7. ALDH Assay

ALDH analysis of breast cancer stem cells was performed using ALDEFLUOR™ Kit (STEMCELL Technologies, Vancouver, Canada). The 2 × 10^5^ single cells /mL were incubated for 30 min at 37 °C in the medium containing the ALDEFLUOR substrate with or without the addition of DEAB. After centrifugation at 1000 rpm for 5 min, the cell pellet was resuspended in 0.5 mL of ALDEFLUOR™ Assay Buffer (STEMCELL Technologies), followed by FACS analysis. These data were analyzed using FlowJo software V10, https://www.bdbiosciences.com/en-us/products/software/flowjo-v10-software (accessed on 10 January 2023).

### 2.8. CD24−/CD44+ Assay

Single-cell suspension in PBS was incubated with PE-conjugated anti-CD24 (12-0247-42, Invitrogen) and FITC-conjugated anti-CD44 (11-0441-81, Invitrogen) in a dark humidity chamber for 30 min, followed by PBST-washing twice for 5 min each time. FACS analysis was performed by flow cytometry (BD Biosciences, Franklin Lakes, NJ, USA).

### 2.9. Mammosphere Assay

Cancer cells were cultured in 6-well ultra-low attachment plates (Corning, New York, NY, USA) with serum-free DMEM/F12 medium containing 1× B27 supplement (Invitrogen), 20 ng/mL human EGF (epidermal growth factor, Sigma) and 20 ng/mL human bFGF (basic fibroblast growth factor, R&D Systems). Each well had 3000 cells. Mammospheres were photographed on days 0, 3, 7 and 10, respectively. Mammospheres with a diameter greater than 40 mm were applied for quantitative analysis.

### 2.10. Western Blot Assay

Primary antibodies (1:1000) include: OCT4 (2750S, Cell Signaling Technology, Danvers, MA, USA), h-TERT (sc-377511, Santa Cruz, CA, USA), SOX2 (3579s, Cell Signaling Technology), NANOG (4903S, Cell Signaling Technology), KLF4 (ARG 55811, Arigo), OCT4-PG1 (ab230429, Abcam, Cambridge, MA, USA), P27 (sc-1641, Abcam), α-Tubulin (ab-7291, Abcam), β-actin (sc-47778, Santa Cruz) and GAPDH (sc-47724, Santa Cruz). HRP-linked anti-rabbit IgG (7074S, Cell Signaling Technology) and HRP-linked anti-mouse IgG (7076S, Cell Signaling Technology) were used as secondary antibodies (1:10,000). All the whole western blot figures can be found in the Supplementary Materials.

### 2.11. Mammary Gland Tumor Burden Mice

For the mammary tumor mice experiment, 1 × 10^7^ MCF-7 cells/mouse overexpressing CCAT2 or control were mixed with Matrigel for cancer cell transplantation. Cells were injected into the fat pad of the fourth mammary gland. The tumor volumes were measured every 7 days from the second week. Six weeks after cell transplantation, the mice were euthanized, and the tumors were taken out for further analysis.

### 2.12. Separation of Cytoplasm and Nucleus

Cells were suspended in buffer A containing 10 mM HEPES pH 7.9, 10 mM KCl, 0.1 mM EDTA, 0.1 mM EGTA, 1 mM dithiothreitol (DTT), 0.15% NP40 and 1% proteinase inhibitor cocktail for 10 min in ice, and centrifuged at 12,000 rpm for 5 min. The supernatant fraction was collected as a cytoplasmic fraction. The pellet was washed with PBS, resuspended in buffer B (20 mM HEPES pH 7.9, 400 mM NaCl, 1 mM EDTA, 1 mM EGTA, 1 mM dithiothreitol (DTT), 0.5% NP40 and 1% proteinase inhibitor cocktail for 15 min of shaking at 4 °C. After centrifugation at 12,000 rpm for 5 min, the supernatant was collected as the nuclear fraction.

### 2.13. Statistical Analysis

GEPIA2 online tool was used for bioinformatics analysis of the TCGA dataset of breast cancer patients. Statistical analyses were performed by using the standard two-tailed *t*-test and one-way ANOVA, in which *p* ≤ 0.05 was considered as statistical significance.

## 3. Results

### 3.1. Downregulation of CCAT2 in Luminal Subtype of Breast Cancer

**Figure 1:**
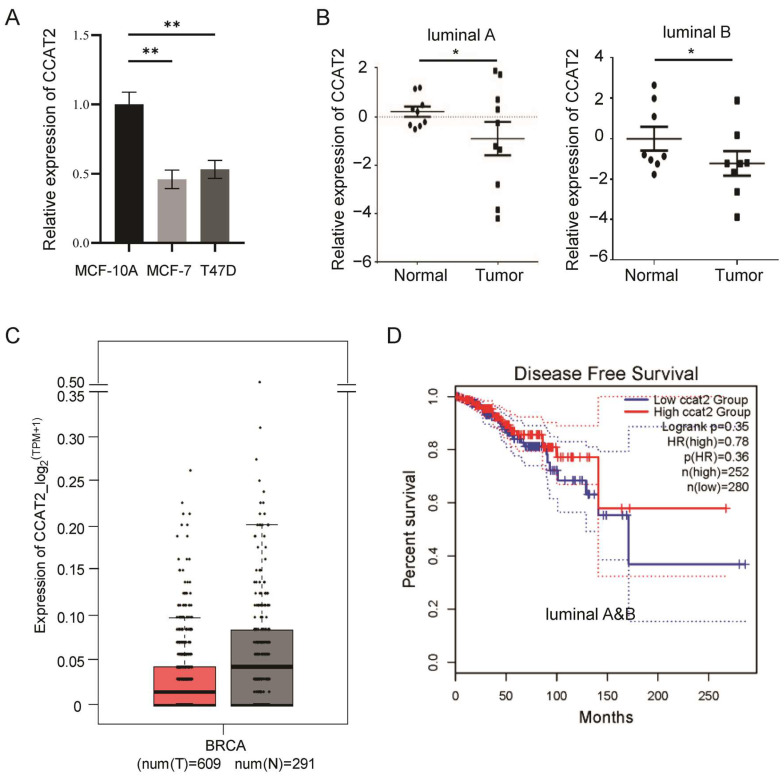
Downregulation of CCAT2 in luminal subtype of breast cancer. (**A**) Lower expression of CCAT2 in two luminal subtype of breast cancer cell lines MCF-7 and T47D, compared to MCF-10A cells. (**B**) Downregulation of CCAT2 in both luminal A and luminal B breast tumor samples, compared to matched adjacent normal mammary tissues (*n* = 10 for each subtype). (**C**) Downregulation of CCAT2 in 609 luminal breast cancer tumors in TCGA database, compared to normal controls (*n* = 609 for tumors and 291 for control). (**D**) A positive correlation between disease-free survival and CCAT2 expression levels in patients with luminal breast cancer. * *p* < 0.05, ** *p* < 0.01.

In order to determine the expression pattern of CCAT2 in the luminal subtype of breast cancer, we analyzed CCAT2 in cancer cell lines and patients’ tumor samples. Compared to normal human mammary gland epithelial cell MCF-10A cell, CCAT2 showed downregulation in two luminal breast cancer cell lines MCF-7 and T47D (Figure 1A). Consistently, a decrease in CCAT2 was observed in both luminal A and luminal B breast tumor samples, compared to matched adjacent normal mammary tissues (Figure 1B), which was further confirmed in 609 luminal breast cancer tumors from the TCGA database (Figure 1C). Meanwhile, a correlation analysis between the patients’ survival and CCAT2 expression indicated better disease-free survival in those CCAT2^high^ patients with luminal breast cancer (Figure 1D). However, the patients with higher levels of CCAT2 showed a trend of worse disease-free survival and worse overall survival in TNBC patients (Supplemental Figure S1A,B), which is consistent with our previous report about the upregulation of CCAT2 in TNBC [25].

### 3.2. Transient Overexpression of CCAT2 Inhibited Cell Proliferation in Luminal Subtype of Breast Cancer

**Figure 2:**
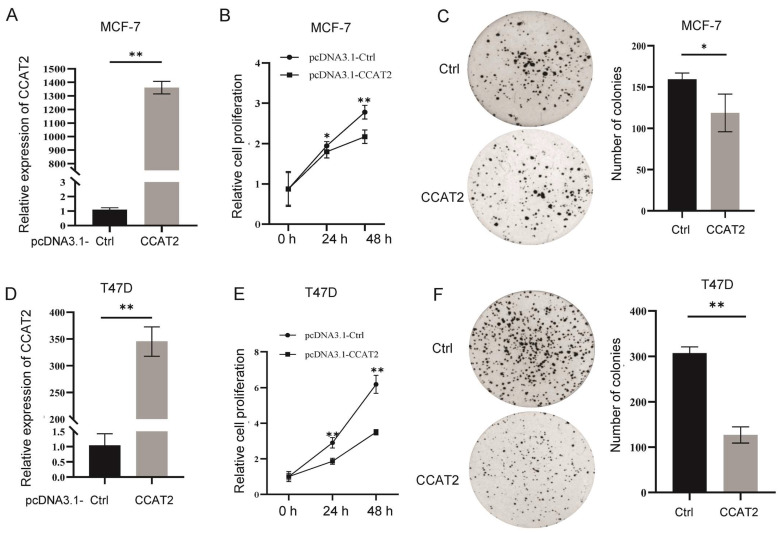
Transient overexpression of CCAT2 inhibited cell proliferation in luminal subtype of breast cancer. (**A**) Overexpression of CCAT2 in MCF-7 cells using pcDNA3.1. (**B**,**C**) pcDNA3.1-mediated overexpression of CCAT2 inhibited cell proliferation (**B**) and colony formation (**C**) in MCF-7 cells. (**D**) pcDNA3.1-mediated overexpression of CCAT2 in T47D cells. (**E**,**F**). pcDNA3.1-mediated overexpression of CCAT2 inhibited cell proliferation (**E**) and colony formation (**F**) in T47D cells. Data are presented as the mean ± SEM (*n* = 3). * *p* < 0.05, ** *p* < 0.01.

In order to determine the function of CCAT2 in luminal breast cancer cells, we transiently overexpressed CCAT2 using pcDNA3.1 plasmid in MCF-7 or T47D cells, followed by cell proliferation analysis and colony formation assay. As shown in Figure 2A–C, pcDNA3.1-mediated overexpression of CCAT2 inhibited cell proliferation and colony formation in MCF-7 cells. Similar results were observed in another luminal breast cancer cell line, T47D (Figure 2D–F).

### 3.3. Transient Transfection of CCAT2 Inhibited Cancer Cell Stemness in Luminal Subtype of Breast Cancer

**Figure 3:**
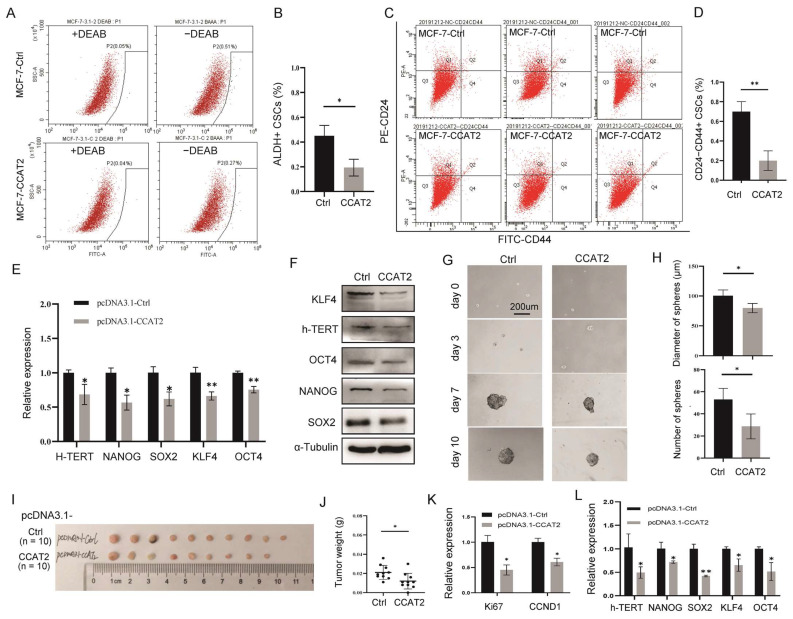
Transient transfection of CCAT2 inhibited cancer cell stemness and suppressed tumor growth in vivo. (**A**) ALDH+ CSC analysis in MCF-7 cells with or without pcDNA3.1-mediated overexpression of CCAT2. DEAB was used in the negative controls of ALDH assay. (**B**) Quantitative analysis of ALDH+ CSCs in (**A**). (**C**) CD24/CD44 analysis in MCF-7 cells with or without pcDNA3.1-mediated overexpression of CCAT2. (**D**) Quantitative analysis of CD24−CD44+ CSCs in (**C**). (**E**,**F**) Downregulation of stemness genes h-TERT, NANOG, SOX2, KLF4 and OCT4 at both mRNA (**E**) and protein (**F**) levels by pcDNA3.1-mediated overexpression of CCAT2 in MCF-7 cells. (**G**,**H**) Mammosphere assays in MCF-7 cells with or without pcDNA3.1-mediated overexpression of CCAT2. (**I**) Mammary tumor mouse models by transplantation of pcDNA3.1-CCAT2-MCF-7 cells or controls demonstrated inhibition of tumor growth by pcDNA3.1-CCAT2 (*n* = 10 in each group, tumors were developed in all of the 10 mice in control group, but in only 9 of the 10 mice in CCAT2 group). (**J**) Tumor weights in (**I**). (**K**) pcDNA3.1-CCAT2 decreased the levels of Ki67 and CCND1 in the MCF-7 cell-derived tumors (*n* = 3). (**L**) pcDNA3.1-CCAT2 suppressed the expression of stemness genes h-TERT, NANOG, SOX2, KLF4 and OCT4 in the MCF-7 cell-derived tumors (*n* = 3). Data are presented as the mean ± SEM. * *p* < 0.05, ** *p* < 0.01.

In view of the CSC regulation by CCAT2 in TNBC [25], we applied a series of cell stemness assays in MCF-7 and T47D cells with or without transient overexpression of CCAT2. We first detected the CSC population in MCF-7 cells overexpressing CCAT2. ALDH+ or CD24^−^CD44+ subpopulations have been identified as breast CSCs [26,27]. Upon transient overexpression of CCAT2, ALDH+ CSCs decreased from ~0.5% to ~0.2% (Figure 3A,B), and CD24^−^CD44^+^ CSCs decreased from ~0.7% to ~0.3% (Figure 3C,D). Gene expression analysis demonstrated downregulation of the stemness genes h-TERT, NANOG, SOX2, KLF4 and OCT4 at both mRNA (Figure 3E) and protein (Figure 3F) levels. Mammosphere assays further confirmed the inhibition of survival and self-renewal of CSCs after transient overexpression of CCAT2 in MCF-7 (Figure 3G,H). Similarly, suppressions of the CSC population, stemness gene expression and mammosphere formation ability by transient overexpression of CCAT2 were further validated in T47D cells (Supplemental Figure S2A–E).

In order to determine the role of CCAT2 in regulating luminal mammary tumor initiation and tumor growth in vivo, the pcDNA3.1-mediated overexpression of CCAT2 in MCF-7 cells was transplanted into the fat pad of immunodeficient female nude mice, followed by tracking of the tumor growth. As shown in Figure 3I, mammary tumors were developed in the mice. Furthermore, pcDNA3.1-mediated overexpression of CCAT2 in MCF-7 cells significantly inhibited the tumor growth in vivo (Figure 3J), decreased the levels of Ki67 and CCND1 (Figure 3K) and suppressed the expression of stemness genes (Figure 3L). These results were consistent with the results in vitro (Figure 2).

### 3.4. Cytoplasmic Distribution and Interaction with miR-221/222 of CCAT2 after Transient Transfection in MCF-7 Cells

**Figure 4:**
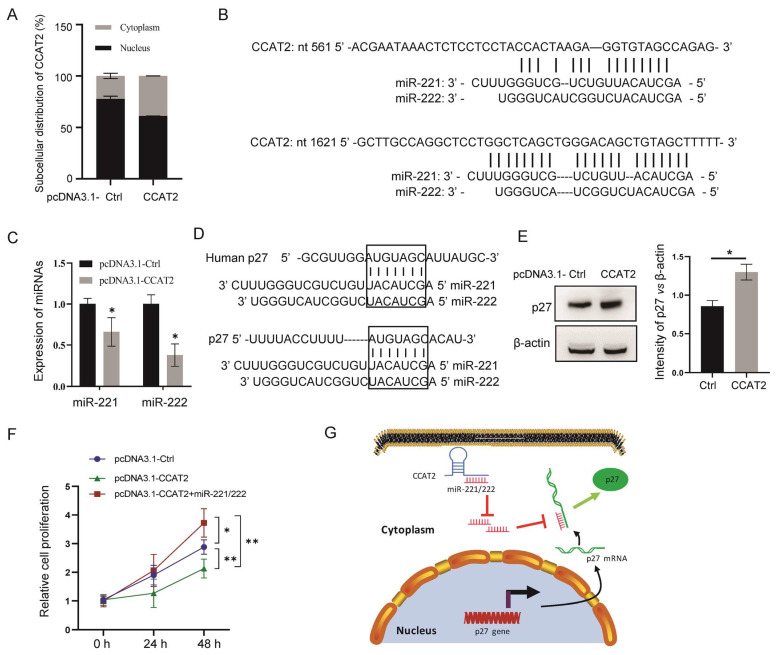
Cytoplasmic localization and interaction with miR-221/222-p27 of CCAT2 after pcDNA3.1-mediated transfection in MCF-7 cells. (**A**) Cytoplasmic distribution of exogenous CCAT2 in MCF-7 cells after pcDNA3.1-mediated transfection. (**B**) BLAST analysis of CCAT2 identified two binding sequences to miR-221/222. (**C**) Downregulation of both miR-221 and miR-222 by cytoplasmic CCAT2. (**D**) Two binding sites to miR-221/222 in mRNA of p27. (**E**). Upregulation of p27 by cytoplasmic CCAT2 in MCF-7 cells. (**F**). Addition back of miR-221/222 mimics into the pcDNA3.1-CCAT2-transfected MCF-7 cells rescued cell proliferation. (**G**) Schematic representation of the regulatory pathway through which miR-221/222-p27 signaling mediated the tumor suppression function of CCAT2 in the cytoplasm. These data are presented as the mean ± SEM (*n* = 3). * *p* < 0.05, ** *p* < 0.01.

Our previous work found a greater distribution of endogenous CCAT2 in the cytoplasm in the luminal subtype of breast cancer cells than that in TNBC cells [28]. Here, we detected the subcellular distribution of exogenous CCAT2 in MCF-7 cells after transfection. As shown in Figure 4A, the cytoplasmic CCAT2 increased from ~20% to ~35% after pcDNA3.1-mediated transfection. Gene BLAST (basic local alignment search tool) analysis of CCAT2 identified two miR-221/222 binding sites at nt581-nt597 and nt1636-nt 1659 (Figure 4B), suggesting the potential of CCAT2 to sponge miR-221/222 as a ceRNA in the cytoplasm of cells. To validate this hypothesis, we analyzed the expression change of miR-221/222 before and after CCAT2 accumulation in the cytoplasm. As a result, both miR-221 and miR-222 showed downregulation by cytoplasmic CCAT2 in MCF-7 cells (Figure 4C). Since miR-221/222 can interact with the mRNA of p27 (Figure 4D), thereby decreasing the expression level of p27 in breast cancer cells [29], we further analyzed the expression of p27 before and after transfection of CCAT2 in MCF-7 cells. As expected, upregulation of p27 was observed after transfection of pcDNA3.1-CCAT2 (Figure 4E). In order to further demonstrate the miRNA signaling to mediate the CCAT2-regulated cell proliferation, an miR-221/222 rescue experiment was performed. As shown in Figure 4F, the addition of miR-221/222 reversed the cell proliferation inhibition by pcDNA3.1-CCAT2. These results suggest that miR-221/222-p27 signaling mediates the tumor suppression function of CCAT2 in the cytoplasm of MCF-7 cells (Figure 4G).

### 3.5. Nuclear Localization of CCAT2 Showed an Oncogenic Function

**Figure 5:**
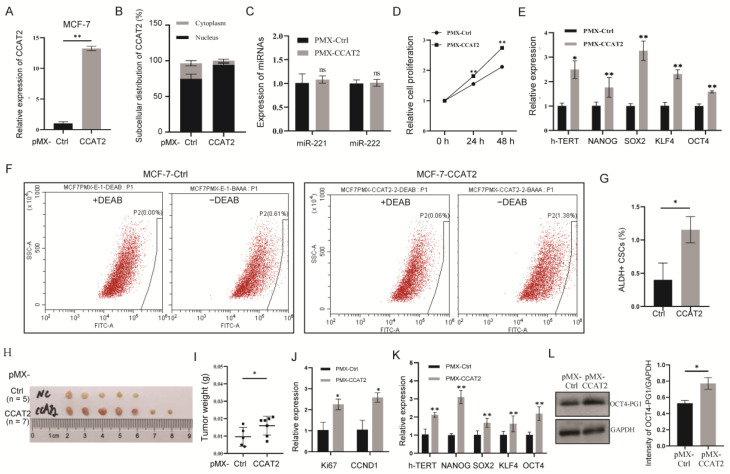
Oncogenic function of CCAT2 in the nucleus of luminal breast cancer. (**A**) Overexpression of CCAT2 in MCF-7 cells using pMX retrovirus. (**B**). Nuclear localization of exogenous CCAT2 in MCF-7 cells after infection using pMX vector. (**C**) Quantitative analysis of miR-221 and miR-222 in MCF-7 cells with or without infection with pMX-CCAT2. (**D**) Induction of cell proliferation by nuclear CCAT2 in MCF-7 cells after infection with pMX-CCAT2. (**E**) Upregulation of stemness genes h-TERT, NANOG, SOX2, KLF4 and OCT4 by nuclear CCAT2 in MCF-7 cells. (**F**) ALDH+ CSC analysis in MCF-7 cells overexpressing pMX-CCAT2 or control. DEAB was used in the negative controls of ALDH assay. (**G**) Quantitative analysis of ALDH+ CSCs in F. (**H**) pMX-CCAT2 infection in MCF-7 cells promoted tumor growth in mice after cell transplantation. (**I**) Tumor weights in (**H**). (**J**) pMX-CCAT2 promoted the levels of Ki67 and CCND1 in the mice tumors. (**K**) pMX-CCAT2 induced the expression of stemness genes h-TERT, NANOG, SOX2, KLF4 and OCT4 in the mice tumors. (**L**) pMX-CCAT2 infection in MCF-7 cells induced the expression of OCT4-PG1. Data are presented as the mean ± SEM (*n* = 3). * *p* < 0.05, ** *p* < 0.01, ns means non-significant.

In order to further determine the function of CCAT2 in the nucleus of the luminal subtype of breast cancer, a pMX retrovirus vector was applied to introduce exogenous CCAT2 into the nucleus of MCF-7 cells (Figure 5A,B), followed by functional analyses. In contrast to the cytoplasmic distribution of CCAT2 after pcDNA3.1-mediated overexpression, stable overexpression of CCAT2 using pMX maintained exogenous CCAT2 in the nucleus of MCF-7 cells, which did not change the expression of miR-221/222 (Figure 5C). Nuclear CCAT2 increased cell proliferation (Figure 5D), promoted the expression of stemness genes (Figure 5E) and induced ALDH+ CSCs from ~0.5% to ~1.2% (Figure 5F,G). Similar results were observed in T47D cells (Supplemental Figure S3A–C).

In addition, a tumor-burden mice model by cell transplantation further demonstrated the promotion of tumor growth by pMX-CCAT2 in vivo (Figure 5H,I), which was associated with increased levels of cell proliferation factors (Ki67 and CCND1, Figure 5J) and cell stemness genes (h-TERT, NANOG, SOX2, KLF4 and OCT4, Figure 5K). A preliminary mechanism study identified a pseudogene of stem cell marker OCT4, OCT4-PG1, as a chromosomally adjacent gene of CCAT2. OCT4-PG1 showed induction by nuclear CCAT2 in luminal breast cancer cells (Figure 5L, Supplemental Figure S4), consistent with our previous report that CCAT2 induced cancer cell stemness in TNBC partly through promoting OCT4-PG1 expression [25].

## 4. Discussion

Although CCAT2 has been reported to promote tumorigenesis in multiple types of cancer [25,30], low expression of CCAT2 in estrogen receptor α (ERα) positive luminal subtype of breast cancer tumors was observed in [24] and our current study. Moreover, a dual function of CCAT2 in regulating tumorigenesis and cancer cell stemness in luminal breast cancer was determined in the current study, in which the cytoplasmic CCAT2 interacted with miRNA 221/222 to interfere with p27-dependent cell proliferation, while CCAT2 accumulation in the nucleus interacted with a pseudogene of OCT4 (OCT4-PG1) to induce cancer stem cells.

For the translational application of CCAT2 to treat breast cancer patients in the future, a drug or gene delivery system to specific subcellular compartments will be required to enrich CCAT2 in the cytoplasm, knockdown CCAT2 in the nucleus, or induce translocation of CCAT2 from nucleus to cytoplasm, thereby playing tumor-suppressing roles as a therapeutic target. An RNA sequence can be either encapsulated in lipid vesicles or conjugated to membrane-penetrating peptides for cytoplasmic delivery. For example, conjugation with Tat or VP22 has been reported to enable the cytosolic delivery of anticancer drugs, siRNAs, plasmid DNA and proteins [31]. Stable nucleic acid-lipid particles or interfering nanoparticles have been designed and applied for the cytoplasmic delivery of siRNAs both in vitro and in vivo. To target the nucleus, SnoVector and pMX vector were verified in [32] and our current study to achieve the nucleic delivery of lncRNAs. In addition to viral-mediated gene delivery, non-viral vectors, chemicals, and peptides have been well-designed to mediate gene transfer to the nucleus. For example, peptides with either amino side chains or guanidinium side chains can penetrate cells through a structure containing five or six glycine residues [33].

As a hormone-activated transcription factor, ERα promotes the expression of various genes that regulate cell proliferation and tumor growth in breast cancer [34]. The subcellular distribution and expression level of lncRNAs in breast cancer cells may be related to the status of ERα. For example, knockdown lncRNA152 and lncRNA67 suppressed cell proliferation in ERα+ luminal breast cancer cell MCF-7 but not in ERα- breast cancer cells [35]. Upregulation of lncRNA H19 was reported to associate with increased ERα expression in endocrine therapy-resistant breast cancer patients. Knockdown of H19 was found to provide an alternative therapeutic strategy for ERα+ drug-resistant breast cancer [36,37,38].

In addition, the current study found that the subcellular distribution of exogenous RNAs may be related to the carrier and/or transfection approach. Upon transfection, a donor gene can locate in the host cell’s cytoplasm, nucleus, mitochondrion, or other organelles to play different roles. This has been widely recognized and well-reviewed for lncRNAs [18,39]. For example, lncRNA thymopoietin antisense transcript 1 (TMPO-AS1) is located in both the cytoplasm and the nucleus of ERα+ breast cancer cells to promote cell proliferation and viability [40]. Linc00839 is located in the nucleus of breast cancer cells, promoting cell proliferation, invasion and migration [41]. The lncRNA MALAT1 is located in the mitochondria of hepatoma cells, regulating the energy metabolism of cancer cells and affecting tumor phenotype [42]. Herein, we found the cytoplasmic distribution of CCAT2 after pcDNA3.1-mediated transfection in luminal breast cancer cells, while pMX-mediated stable overexpression of CCAT2 maintained CCAT2 in the nucleus. It remained an open question of how pcDNA3.1-mediated transfection translocated CCAT2 from the nucleus to the cytoplasm. This is being addressed in our continued work. As stated by Bridges MC, et al., in a recent review [18], “subcellular localization of lncRNAs is an additional essential layer of complexity that is required to be taken into account to fully understand the roles of lncRNAs in any cellular function”. The review article summarized current knowledge of lncRNA subcellular localization and factors controlling their localization, including sequence motifs, ribosome association, interaction with RNA-binding partners, insufficient splicing, the secondary structure and posttranscriptional modifications of lncRNAs [18].

Collectively, these findings in the current study about the subcellular distribution-related function of lncRNAs will lead to a better understanding of non-coding RNA involvement in cancer regulation and more precise medical application of novel therapeutic targets in the treatment of breast cancer.

## Data Availability

All data are included in this published article and Supplementary Files. All materials we generated ourselves are shareable upon request.

## Supplementary Materials

The following supporting information can be downloaded at: https://www.mdpi.com/article/10.3390/cancers15020538/s1, Figure S1: Correlation between CCAT2 levels and disease-free survival (A) and overall survival (B) in TNBC patients; Figure S2: A. ALDH+ CSC analysis in T47D cells with or without pcDNA3.1-mediated overexpression of CCAT2. DEAB was used in the negative controls of ALDH assay. B. Quantitative analysis of ALDH+ CSCs in A. C. Downregulation of stemness genes h-TERT, NANOG, SOX2, KLF4 and OCT4 by pcDNA3.1-mediated overexpression of CCAT2 in T47D cells. D. Mammosphere assays in T47D cells with or without pcDNA3.1-mediated overexpression of CCAT2. E. Quantitative analysis of the number and average diameter of the spheres in D. Data are presented as the mean ± SEM (*n* = 3). * *p* < 0.05, ** *p* < 0.01; Figure S3: Oncogenic function of pMX-CCAT2 in T47D cells. A. Induction of cell proliferation by pMX-CCAT2 in T47D cells. B. Upregulation of stemness genes h-TERT, NANOG, SOX2, KLF4 and OCT4 by pMX-CCAT2 in T47D cells. C. Promotion of ALDH+ CSCs in T47D cells after infection with pMX-CCAT2. DEAB was used in the negative controls of ALDH assay. Data are presented as the mean ± SEM (*n* = 3). * *p* < 0.05, ** *p* < 0.01; Figure S4: Original blots for western blot images of Figure 3F, Figure 4E and Figure 5L.
