# Supplementary material for: Dual Function of CCAT2 in Regulating Luminal Subtype of Breast Cancer Depending on the Subcellular Distribution
Source: Cancers (Basel). 2023 Jan 16;15(2):538. doi: 10.3390/cancers15020538 (PMC9856762; doi:10.3390/cancers15020538)

Dual function of CCAT2 in regulating luminal subtype of breast cancer depending on the subcellular distribution

Supplemental Figure S1: Correlation between CCAT2 levels and disease-free survival (A) and overall survival (B) in triple negative breast cancer (TNBC) patients.

Supplemental Figure S2: A. ALDH+ CSC analysis in T47D cells with or without pcDNA3.1-mediated overexpression of CCAT2. DEAB was used in the negative controls of ALDH assay. B. Quantitative analysis of ALDH+ CSCs in A. C. Downregulation of stemness genes h-TERT, NANOG, SOX2, KLF4 and OCT4 by pcDNA3.1-mediated overexpression of CCAT2 in T47D cells. D. Mammosphere assays in T47D cells with or without pcDNA3.1-mediated overexpression of CCAT2. E. Quantitative analysis of the number and average diameter of the spheres in D. Data are presented as the mean ± SEM (n=3). *p<0.05, **p<0.01.

Supplemental Figure S3: Oncogenic function of pMX-CCAT2 in T47D cells. A. Induction of cell proliferation by pMX-CCAT2 in T47D cells. B. Upregulation of stemness genes h-TERT, NANOG, SOX2, KLF4 and OCT4 by pMX-CCAT2 in T47D cells. C. Promotion of ALDH+ CSCs in T47D cells after infection with pMX-CCAT2. DEAB was used in the negative controls of ALDH assay. Data are presented as the mean ± SEM (n=3). *p<0.05, **p<0.01.

Supplemental Figure S4: Original blots for western blot images of Figure 3F, 4E and 5L.

Supplemental Figure S 1

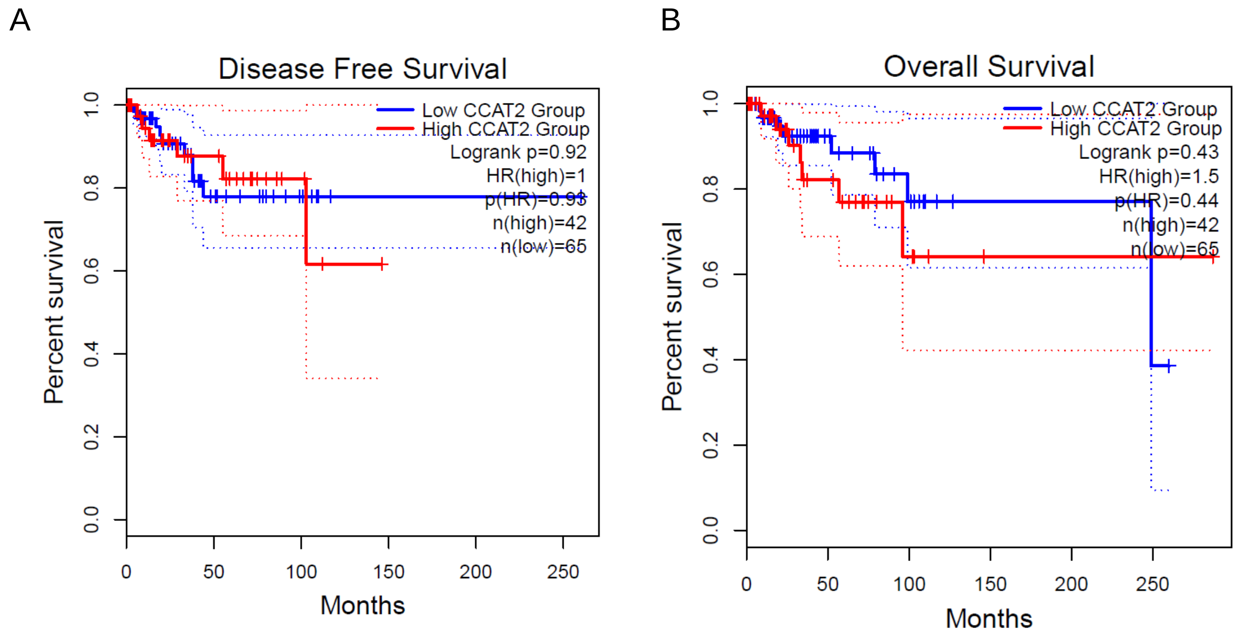

Supplemental Figure S 2

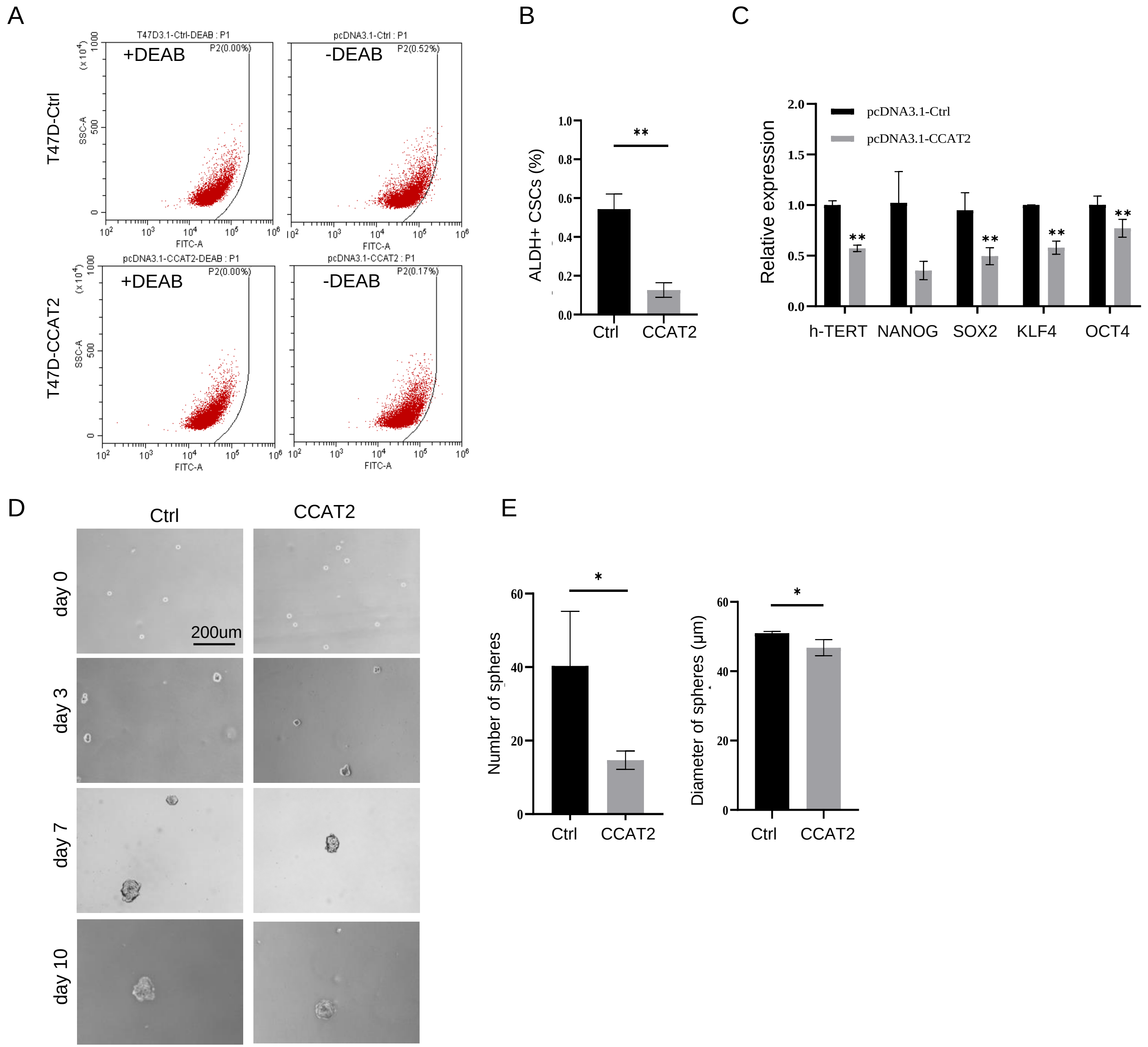

Supplemental Figure S3

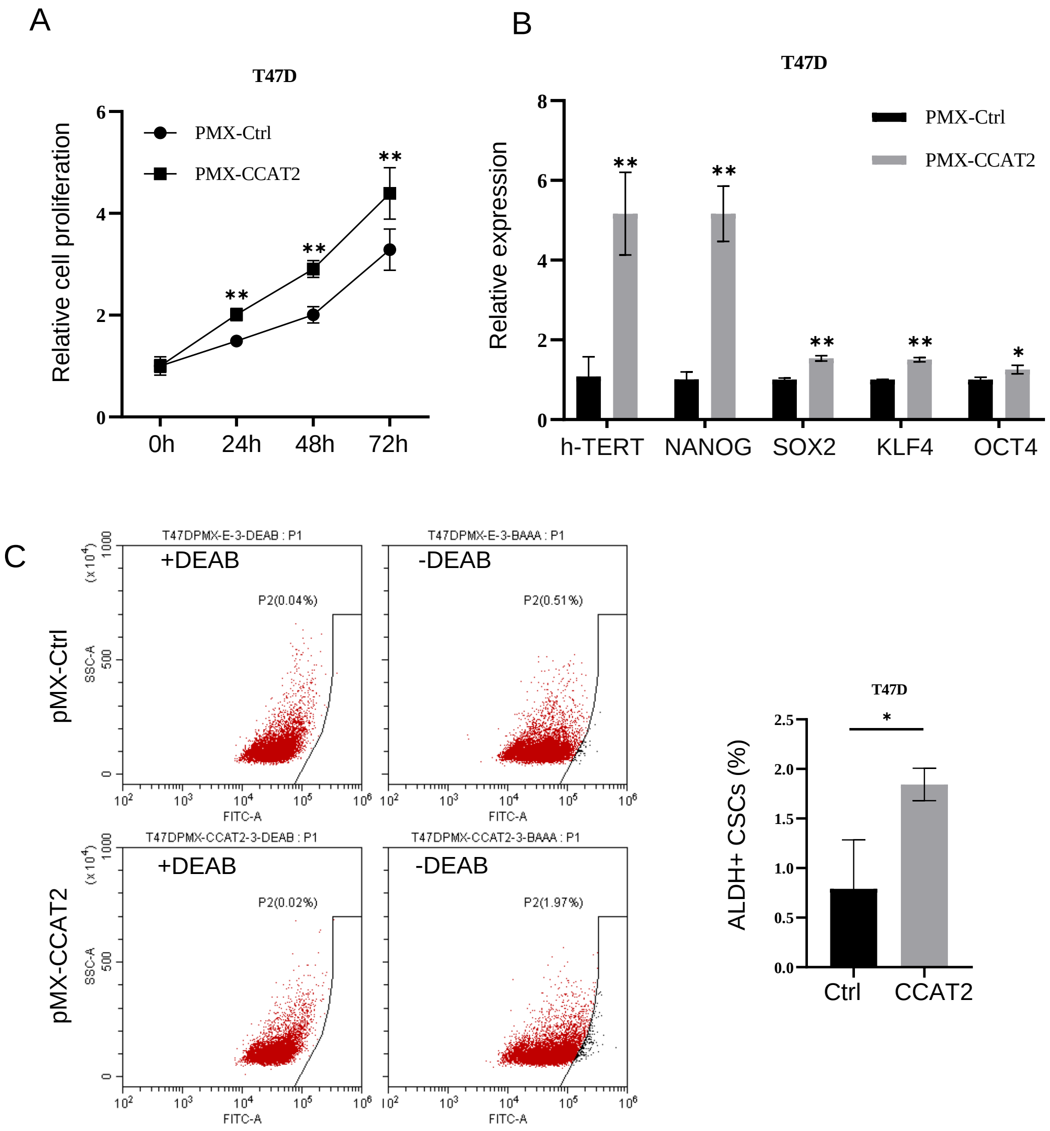

Supplemental Figure S4

A: Original blots for Figure 3F

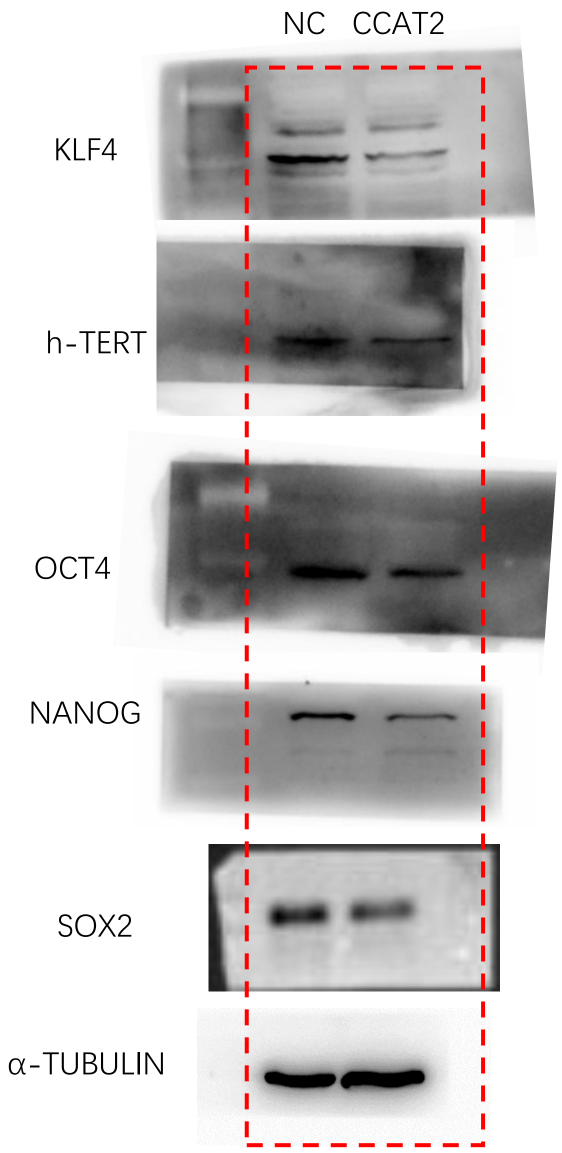

B: Original blots for Figure 4E

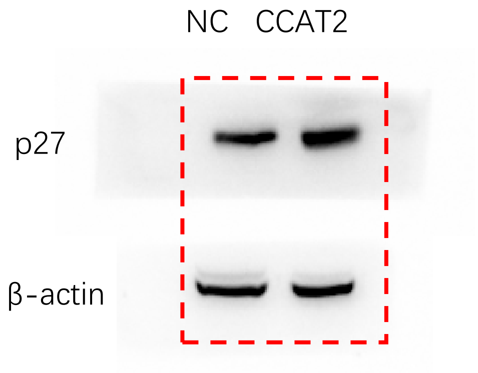

C: Original blots for Figure 5L

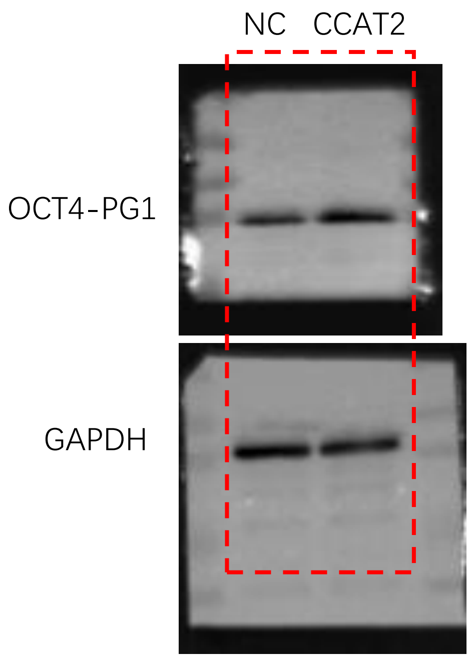

Supplement: Supplementary file 1 [file cancers-15-00538-s001.zip › cancers-2157008-supplementary.pdf]
